# PSYCHOLOGICAL WELL-BEING AND PHYSICAL HEALTH ON SUBJECTIVE AND OBJECTIVE MEMORY IN OLDER ADULTS

**DOI:** 10.1093/geroni/igac059.2068

**Published:** 2022-12-20

**Authors:** Zhong-xu Liu, Brenda Whitehead, Anda Botoseneanu

**Affiliations:** University of Michigan Dearborn, Dearborn, Michigan, United States; Grace College, Winona Lake, Indiana, United States; University of Michigan Dearborn, Dearborn, Michigan, United States

## Abstract

Self-report measures of memory, often used in survey studies of older adults, are heavily influenced by stress, depression, and life satisfaction; this makes it difficult to tease memory performance apart from psychological well-being, and highlights the value of shifting to remotely-administered objective memory tasks when feasible. This study investigates how indicators of psychological well-being, psychological distress, and physical health differentially influence the subjective and objective memory measures in order to compare the extent to which they are explained by participants’ psychological and physical health profile. 404 adults aged 55 and older without diagnosed cognitive impairment participated in an online survey which involved measures of physical health (PHY; multimorbidity, BMI), psychological well-being (PWB; life satisfaction, positive and negative affect), psychological distress (PDS; perceived stress, anxiety, depression) and subjective memory complaints (SM), along with remotely-administered objective memory tasks (OM). Regression analyses found all three health/well-being composite variables (PHY, PWB, PDS) maintained significant effects on SM (p < .01); PWB and PDS had no significant effects on OM, whereas PHY maintained significance on OM throughout (p = .02).So, SM measures are highly influenced by the psychological profile of the participant, highlighting the importance of controlling for these factors when relying on subjective memory measures. That physical health was the only significant predictor of the OM tasks in this study not only reveals remotely-administered OM tasks to be more immune to participants’ psychological profile, but also supports previously-established links between physical health and brain function.

